# Protection of Retinal Ganglion Cells and Retinal Vasculature by Lycium Barbarum Polysaccharides in a Mouse Model of Acute Ocular Hypertension

**DOI:** 10.1371/journal.pone.0045469

**Published:** 2012-10-19

**Authors:** Xue-Song Mi, Qian Feng, Amy Cheuk Yin Lo, Raymond Chuen-Chung Chang, Bin Lin, Sookja Kim Chung, Kwok-Fai So

**Affiliations:** 1 Department of Ophthalmology, the First Affiliated Hospital of Jinan University, Guangzhou, Guangdong, China; 2 Department of Anatomy and the State Key Laboratory of Brain and Cognitive Science, Li Ka Shing Faculty of Medicine, The University of Hong Kong, Hong Kong, China; 3 Research Centre of Heart, Brain, Hormone and Healthy Aging, Li Ka Shing Faculty of Medicine, The University of Hong Kong, Hong Kong, China; 4 Eye Institute, Li Ka Shing Faculty of Medicine, The University of Hong Kong, Hong Kong, China; 5 The Joint Laboratory for Brain Function and Health (BFAH), Jinan University and The University of Hong Kong, Hong Kong, China; Dalhousie University, Canada

## Abstract

Acute ocular hypertension (AOH) is a condition found in acute glaucoma. The purpose of this study is to investigate the protective effect of *Lycium barbarum* polysaccharides (LBP) and its protective mechanisms in the AOH insult. LBP has been shown to exhibit neuroprotective effect in the chronic ocular hypertension (COH) experiments. AOH mouse model was induced in unilateral eye for one hour by introducing 90 mmHg ocular pressure. The animal was fed with LBP solution (1 mg/kg) or vehicle daily from 7 days before the AOH insult till sacrifice at either day 4 or day 7 post insult. The neuroprotective effects of LBP on retinal ganglion cells (RGCs) and blood-retinal-barrier (BRB) were evaluated. In control AOH retina, loss of RGCs, thinning of IRL thickness, increased IgG leakage, broken tight junctions, and decreased density of retinal blood vessels were observed. However, in LBP-treated AOH retina, there was less loss of RGCs with thinning of IRL thickness, IgG leakage, more continued structure of tight junctions associated with higher level of occludin protein and the recovery of the blood vessel density when compared with vehicle-treated AOH retina. Moreover, we found that LBP provides neuroprotection by down-regulating RAGE, ET-1, Aβ and AGE in the retina, as well as their related signaling pathways, which was related to inhibiting vascular damages and the neuronal degeneration in AOH insults. The present study suggests that LBP could prevent damage to RGCs from AOH-induced ischemic injury; furthermore, through its effects on blood vessel protection, LBP would also be a potential treatment for vascular-related retinopathy.

## Introduction

Glaucoma, the leading cause of vision loss in the world [1], is associated with the loss of retinal ganglion cells (RGCs) and their axons [2]. Although the elevation of intraocular pressure (IOP) plays a key role in the mechanism of glaucoma, other factors including ischemia [3] are also involved in the pathogenesis. The acute ocular hypertension (AOH) is a well-established animal model for producing retinal degeneration, which has been used to investigate the pathogenesis of RGC death and possible therapeutic interventions for neuroprotection [4], [5], [6].

Previous studies suggest that neurodegeneration in glaucoma undergoes two phases: the direct damage to RGC and axons and the secondary damage by responses of non-neuronal cells. The secondary damage is considered to be the major cause of RGC loss in glaucoma [7], [8]. The breakdown of blood-brain-barrier (BBB) and blood-retinal-barrier (BRB) has been reported in transient middle cerebral artery occlusion (MCAO)-induced ischemic injury in the brain and retina [9], [10], [11], [12]. However, long-term effects of disrupted BRB on retinal ganglion cells and blood vessels have not been reported in AOH retinal injury.

Endothelin-1 (ET-1), synthesized in vascular endothelial cells, is a potent vasoconstrictor. Over-expression of ET-1 could induce BBB damage by down-regulating the level of occludin, the key protein to construct tight junction between blood vessel endothelial cells [10]. RAGE, the receptor for advanced glycation end-products (AGEs), can recognize multiple ligands such as amyloid-β and AGEs. Over-expressed RAGE on blood vessel endothelial cells can activate the membrane-transporting system of AGE-RAGE and Aβ, resulting in accumulation of AGEs and Aβ in parenchyma and release of ET-1, which is reported in diabetic microangiopathy and Alzheimer's disease (AD) [13], [14], [15]. However, their roles in AOH retinal injury still do not define.

*Lycium barbarum*, known as Fructus Lycii or Wolfberry in the West, is a kind of traditional Chinese medicine (TCM) for a very long period of time with anti-aging effects. Interestingly, potential neuroprotective effects of *Lycium barbarum* polysaccharides (LBP) on neurons in the CNS has recently been discovered in many previous studies by different groups [16], [17], [18], [19], [20], [21], [22]. Our previous studies have shown the neuroprotective effects of LBP on RGCs in both a chronic ocular hypertension model of glaucoma [23], [24], [25] and in MCAO-induced ischemic retina [11]. In addition, the protective effects of LBP against Aβ neurotoxicity on neurons in Alzheimer's disease have also been observed recently [18], [19], [20]. In the present study, we want to explore the protective effects of LBP on retinal ganglion cells, blood-retinal-barrier (BRB) and blood vessels in AOH models.

## Methods

### Animals

C57BL/6N male mice (10 to 12 weeks, weight around 20–25 g) were used in this study. They were maintained on a 12 hour light-dark cycle and received food and water *ad libitum*. All experimental designs and protocols were conducted according to the institutional guidelines for the care and use of laboratory animals at The University of Hong Kong, and were approved by the Committee for the Use of Live Animals in Teaching and Research (CULTRA#1664-08) at The University of Hong Kong.

### Animal model of acute ocular hypertension (AOH)

The animals were anesthetized with a mixture of ketamine (80 mg/kg) and xylazine (8 mg/kg). One drop of proparacaine hydrochloride 0.5% (Alcaine; Alcon, Ltd., Fort Worth, TX) followed by a drop of mydriacyl (Alcon, Ltd., Fort Worth, TX) was applied to the eyes for desensitizing the cornea and mydriasis. AOH was induced by inserting a micro-glass needle into the anterior chamber of the right eye. The needle was linked with a reservoir of balance salt solution (BSS, Alcon, Ltd., Fort Worth, TX) to keep the IOP at 90 mmHg for 60 minutes. The state of AOH was verified by checking the whitening of the anterior segment of the globe and blanching of episcleral veins under the surgical microscopy. During the surgical procedure, animal's temperature was kept at 37±0.5°C with a heating pad. After the procedure, 0.3% tobramycin ointment (Alcon, Fort Worth, TX) was applied to the conjunctival sac.

### Drug administration

The preparation for *Lycium barbarum* polysaccharides (LBP) extracts was the same as reported previously [18]. Here, a pre-treatment procedure was used [11], [23], [24]. The freeze-dried powder of LBP was freshly diluted with phosphate-buffered saline (PBS; 0.01 M; pH 7.4). Experimental animals were divided into two groups: orally feed with either LBP solution or PBS as vehicle-treated control (n = 7 per group). Drug administration was performed using a feeding needle with LBP of 1 mg/kg or vehicle daily from 7 days before the insult till sacrifice.

### Sample processing

Animals were sacrificed with an overdose of sodium pentobarbital. Eyeballs were removed and fixed in 4% paraformaldehyde (PFA) overnight at 4°C. Using a suture to clarify the orientation of each eyeball, samples were dehydrated with a graded series of ethanol and xylene and subsequently embedded in paraffin wax. Cross-sections of 7 µm thick were cut. For some animals, retinal flat-mounts were prepared after removing the eye ball. The flat-mounts were fixed in 4% PFA for 2–4 hours.

### Immunohistochemistry

Samples were rinsed with PBS, and blocked in PBS with 0.3% Triton X-100 and 10% goat serum for 1 hour. Then, they were incubated with primary antibodies overnight at 4°C, followed by secondary antibodies (Table 1). For cross-sections, fluorescence secondary antibody was incubated for 2 hour at room temperature, or biotinylated secondary antibody was incubated for 1 hour and then conjugated with avidin-biotin-peroxidase-complex (ABC) kit and detected using Diaminobenzidine tetrahydrochloride (DAB). For flat-mounted retinas, secondary antibody was incubated overnight at 4°C. Diamidino-2-phenylindole (DAPI) 0.2% was used in some staining procedures to visualize the nuclear. Retinal whole mounts were then mounted on slides and visualized using a fluorescent microscope (Zeiss, Oberkochen, Germany) or confocal laser scanning microscope LSM 510 Meta (Zeiss, Oberkochen, Germany).

**Table 1:** Antibody list.

Antibody	Dilution	Host	Company	Cat. Number
β-tubulin III	1∶500	Mouse	Covance	MMS-435P
GFAP	1∶400	Mouse	Sigma	G3893
GS	1∶600	Mouse	Millipore	MAB302
PECAM-1	1∶400	Rat	BD Pharmingen	553370
α-SMA	1∶200	Mouse	Sigma	A2547
NG2	1∶200	Rabbit	Millipore	AB5320
Occludin	1∶1000	Rabbit	Invitrogen	40-4700
ET-1	1∶1600	Rabbit	Peninsula	T-4050
RAGE	1∶200	Rabbit	Abcam	ab3611
Aβ_1–42_	1∶50	Rabbit	Abcam	ab10148
AGE	1∶200	Rabbit	Genetex	GTX43720
Alexa Fluor 568 (Goat anti-rabbit)	1∶200		Molecular Probes	A11036
Alexa Fluor 488 (Goat anti-mouse)	1∶200		Molecular Probes	A11029
Alexa Fluor 568 (Goat anti-rat)	1∶200		Molecular Probes	A11077
DAPI	1∶5000		Sigma	D9564
DAB kit			Invitrogen	002114
MOM kit			Vector Lab	FMK-2201

### IgG extravasations

IgG leakage can be used to detect the damage of the BRB, which has been reported in previous studis [11], [26]. Retinal sections were prepared as described above and blocked with 10% normal goat serum for 1 hour. Then they were incubated with Mouse-On-Mouse biotinated anti-mouse IgG secondary antibody and avidin-biotin-peroxidase-complex (ABC). The positive immunoreactivity of IgG was detected with DAB.

### Histological evaluations

On flat-mounted retinas, RGCs were labeled and counted using β-tubulin III staining [27], [28]. The counting method has been described previously [29], [30]. Briefly, under an eye-piece grid of 200×200 µm^2^ along the median line of each quadrant, six microscopic fields for each quadrant with a total of 24 areas per retina were counted starting from the optic disc to the border at 400 µm intervals, corresponding to approximately 3% of each retina. The data were shown as percentage loss of RGCs.

On retinal cross-sections, several histological evaluations were performed. Hematoxylin and Eosin (H&E) staining was done to visualize the inner retinal layer (IRL), which ranges from the inner limiting membrane (ILM) to the inner nuclear layer (INL). We chose to measure the thickness of IRL because previous studies have reported that the main alteration of retina after AOH is formed in the IRL [31] [32]. Our pilot study also did not find any significant change in the outer nuclear layer (ONL). For consistency, the sections containing optic nerve stump were used, and at least three discontinuous sections (per animal) were analyzed. Image at 400× magnification of the middle retina at 1.1 mm on both sides from the optic nerve head [33] were analyzed using StereoInvestigator® software (MBF Bioscience-MicroBrightField Inc).

For other evaluations (IgG, PECAM-1, α-SMA), at least 10 discontinuous sections (per animal) were randomly chosen to perform fluorescence staining. Quantitative method was used as reported previously [26], [34] [35]. The modification in the present study was that the staining signals were calculated by per millimeter (mm), but not using per square pixel, since the thickness of retinas has been significantly changed in AOH model [31], [32], however the retinal length is constant theoretically. IgG was used to represent the blood vessel density as described by our previous studies [26]. Each dot or tube was counted as one signal of blood vessels. Blood vessel density was estimated by counting all signals in the ganglion cell layer (GCL) and inner nuclear layer (INL) versus the whole length of the retina. PECAM-1 labeled endothelial cells and α-SMA labeled pericytes in capillaries were also counted as described above.

### Western blotting

The level of occludin and RAGE in the retina was measured using Western blotting. The retinas were homogenized in lysis buffer (10 mM Tris pH 7.4, 150 mM NaCl, 1 mM EDTA, 1 mM EGTA, 10% protease inhibitor cocktail and 1% phosphatase inhibitor cocktails) and centrifuged (2000 g, 5 min, 4°C). The supernatant was measured by protein assay kit (Bio-Rad Laboratories, US). A 40 µg aliquot of proteins from each individual animal was subjected to 12.5% SDS-polyacrylamide gel electrophoresis and transferred into PVDF membrane. The blot was incubated with antibody against occludin (1∶1000, Invitrogen), RAGE (1∶1000, R&D) and β-actin (1∶10000, Santa Cruz). The secondary antibody was Horseradish Peroxidase-conjugated secondary antibody (Dako, Japan). Signals were visualized with ECL (Amersham, Buckinghamshire, UK) and quantitated using Image J software. The ratio of the expression of occludin and RAGE was determined after normalizing the individual β-actin levels.

### Statistics

Retinas of the contralateral eyes from the PBS-fed-AOH and LBP-fed-AOH groups formed the non-AOH control group. The PBS-fed-AOH group was compared with non-AOH control group. The LBP-fed-AOH group was compared with PBS-fed-AOH and non-AOH control groups. Each analysis was performed in a blinded manner to eliminate any subjective bias, where the slides were analyzed by observers-blinded to the identification of the mice. Data were presented as mean ± standard deviation (SD). One-way analysis-of-variance (ANOVA) followed by Bonferroni's post test was used for the counting data analysis. Kruskal-Wallis Test followed by the Dunn's multiple comparison tests was used for semi-quantitative analysis of ICC staining intensity using GraphPad Prism software (San Diego, CA, USA). Statistically significant difference was set at *P*<0.05.

## Results

### LBP protected retina from neuronal loss in AOH model

**Figure 1:**
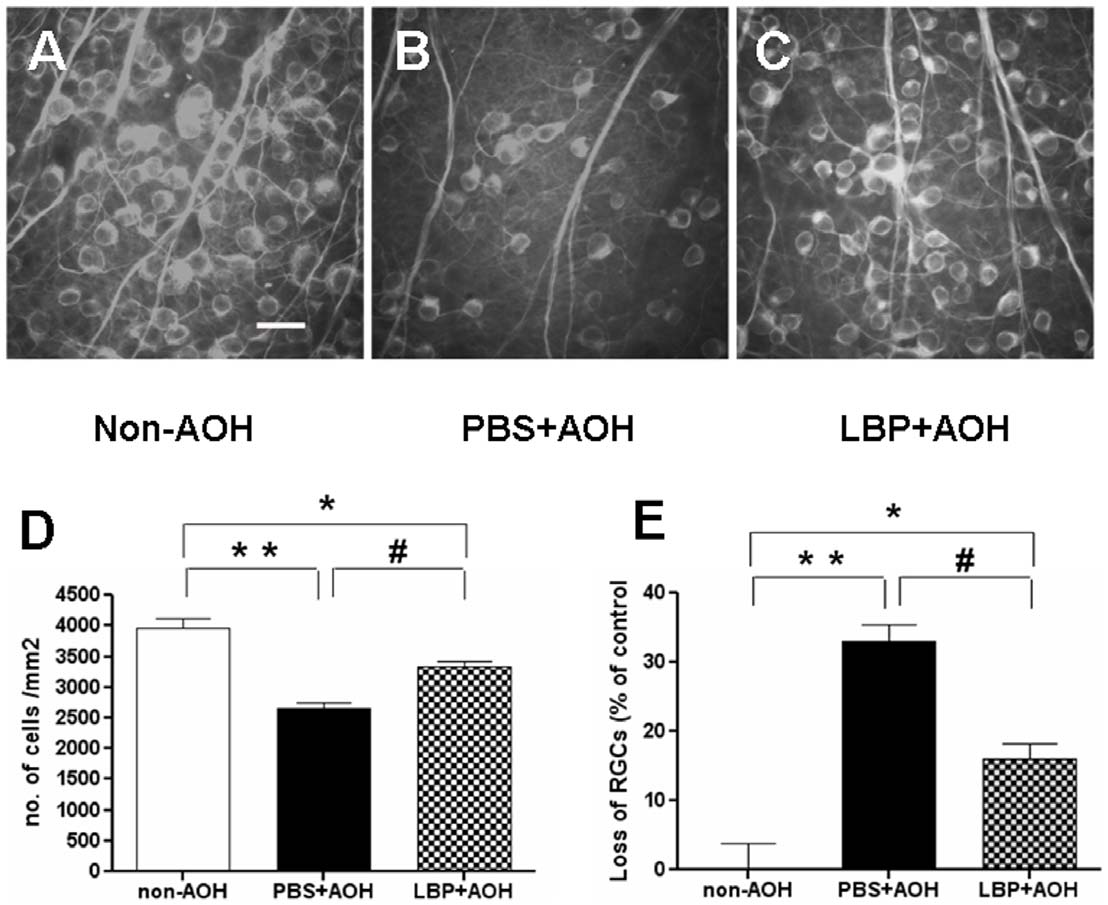
Protection of RGCs in LBP-fed-AOH retina at day 4 after AOH. (A–C) Representative photos of β-tubulin-III labeled RGCs on retinal flatmounts from mid-peripheral area, in the non-AOH control group (A), PBS-fed-AOH group (B) and LBP-fed-AOH group (C). (D) Quantification of no. of cells/mm2. (E) Quantification of the percentage of loss of RGCs. * P<0.05, # P<0.01, ** P<0.001. Scale bar: 20 µm. RGCs, retinal ganglion cells; PBS, phosphate-buffered saline vehicle; AOH, acute ocular hypertension; LBP, *Lycium Barbarum Polysaccharides* solution.

AOH procedure induced an obvious loss of RGCs and thinning of IRL as reported by the previous studies [31], [32]. To detect the effects of LBP, we estimated the number of RGCs at day 4 post the AOH insult and the IRL thickness at day 7 after the operation when the acute damage became stable in the retinal tissue. At day 4 post-operation, the loss of RGCs in the PBS-fed-AOH group was severe (33±2.4%) with remaining RGC number at 2654±98.1/mm^2^ (Figure 1B) when compared with the non-AOH control retina (3962±151.3/mm^2^) (Figure 1A) (P<0.001). Compared with the PBS group, the number of remaining RGCs was higher in the LBP-fed-AOH group (3328±91.6/mm^2^) (Figure 1C) (P<0.01); however, there was still a significant loss of RGCs in the LBP-fed-AOH group (15±2.3%) when compared with the non-AOH control retina (P<0.05) (Figure 1D, 1E).

**Figure 2:**
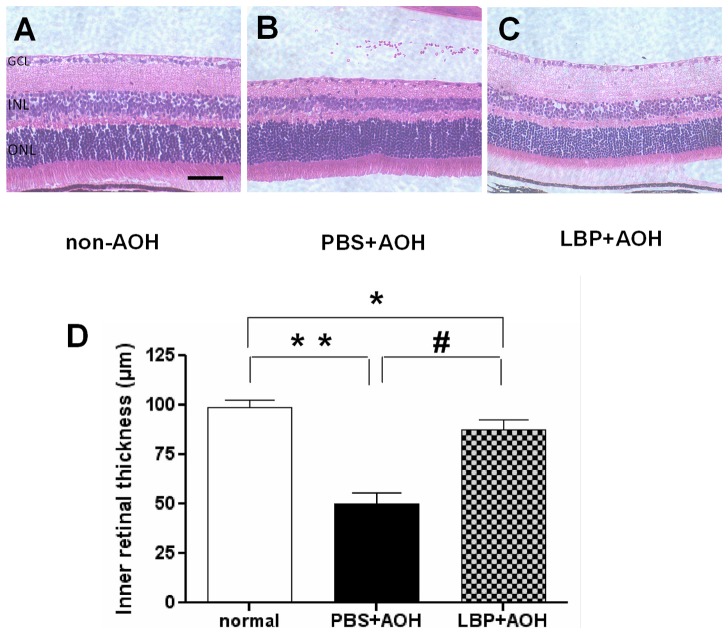
Protection of inner retinal layer thickness in LBP-fed-AOH retina at day 7 after AOH. (A–C) Representative photos of HE-stained retinal sections from the middle area in the non-AOH control group (A), PBS-fed-AOH group (B) and LBP-fed-AOH group (C). (D) Quantification of the inner retinal thickness. * P<0.05, # P<0.01, ** P<0.001. Scale bar: 50 µm. HE, Hematoxylin and Eosin staining; PBS, phosphate-buffered saline vehicle; AOH, acute ocular hypertension; LBP, *Lycium Barbarum Polysaccharides* solution; ILM, internal limiting membrane; INL, inner nuclear layer.

At day 7 post-operation, the IRL was thinnest (50±5.2 µm) in the PBS-fed-AOH group (Figure 2B); it was also thinner in the LBP-fed-AOH group (87±4.9 µm) (Figure 2C) when compared with the non-AOH control retina (98±3.6 µm) (Figure 2A). Statistical analyses showed significant differences among the three groups (Figure 2D).

### LBP protected the stability of retinal vasculature

**Figure 3:**
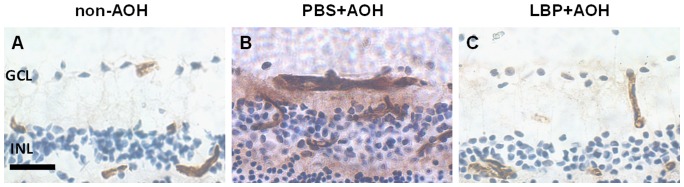
Prevention of BRB leakage in LBP-fed retina at dat 4 after AOH. Representative photos of IgG staining on retinal sections, in the non-AOH control group (A), PBS-fed-AOH group (B) and LBP-fed-AOH group (C). Scale bar: 25 µm. BRB, blood-retinal-barrier; PBS, phosphate-buffered saline vehicle; AOH, acute ocular hypertension; LBP, *Lycium Barbarum Polysaccharides* solution; GCL, ganglion cell layer; INL, inner nuclear layer.

To detect the effects of LBP on the protection of BRB, IgG extravasation was examined using the DAB method on retinal sections. Using this method, the IgG leakage appeared as diffuse and fuzzy non-shaping brown signals distributed in the tissue outside the leaky blood vessels as described in many previous studies [9], [10], [26]. Here, when compared with the non-AOH control (Figure 3A), the IgG staining appeared as dark-brown non-shaping signals outside the leaked blood vessels diffusing in the retinal parenchyma in the PBS-fed-AOH retina (Figure 3B). However, it was difficult to identify which blood vessel contributed to the leakage so we did not count the numbers of leaky blood vessels. In the LBP-fed-AOH group (Figure 3C), the IgG staining in retinal parenchyma appeared lighter than the PBS group, but was also presented as weak fuzzy brown signals around the blood vessels when compared with the non-AOH control (Figure 3A).

**Figure 4:**
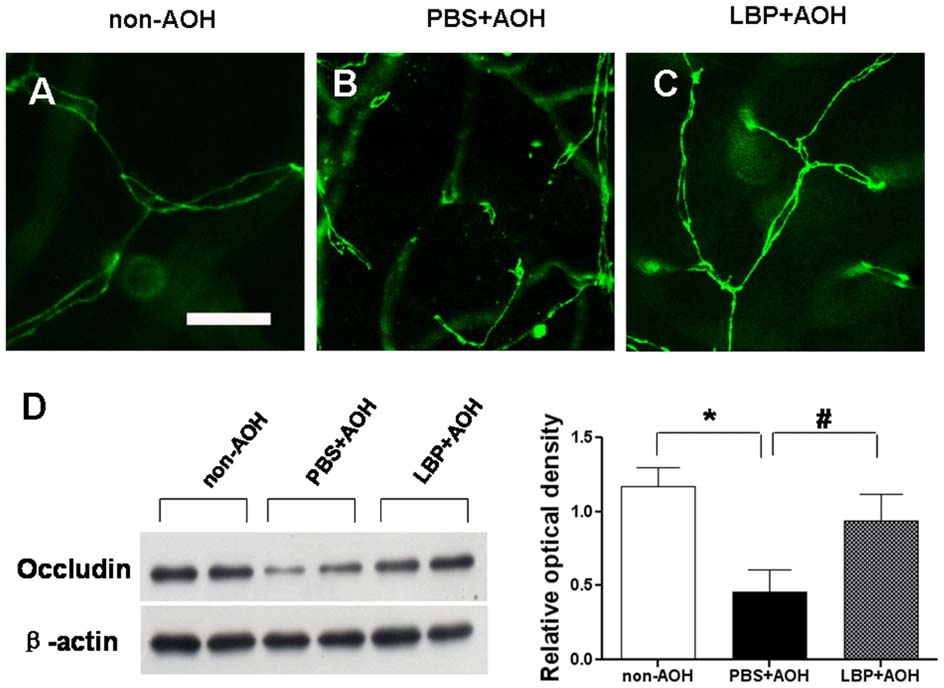
Protection of tight junctions between endothelial cells in LBP-fed retina at day 4 after AOH. (A–C) Representative photos of occludin-stained tight junctions in the network of capillaries on retinal flat-mounts, in the non-AOH control group, PBS-fed-AOH group and LBP-fed-AOH group. Note that the broken chains of tight junctions in (B). Scale bar: 15 µm. PBS, phosphate-buffered saline vehicle; AOH, acute ocular hypertension; LBP, *Lycium Barbarum Polysaccharides* solution.

Using occludin staining, we verified that the change in tight junction integrity between blood vessel endothelial cells might be one of the causes for the IgG leakage from the blood vessels in the AOH model. On retinal flat-mounts, in the network of capillaries in the non-AOH control (Figure 4A), the tight junctions appeared as chains along the capillaries; however, in the PBS-fed-AOH group (Figure 4B), broken tight junctions were observed everywhere in capillaries. On the contrary, in the LBP-fed-AOH group (Figure 4C), the tight junctions were shown as un-broken chains along the capillaries similar to the non-AOH control. Western blotting data confirmed the decreased level of occludin in the PBS-fed-AOH group when compared with the non-AOH control and the increased level in the LBP-fed-AOH group when compared with the PBS-fed-AOH group (Figure 4D).

**Figure 5:**
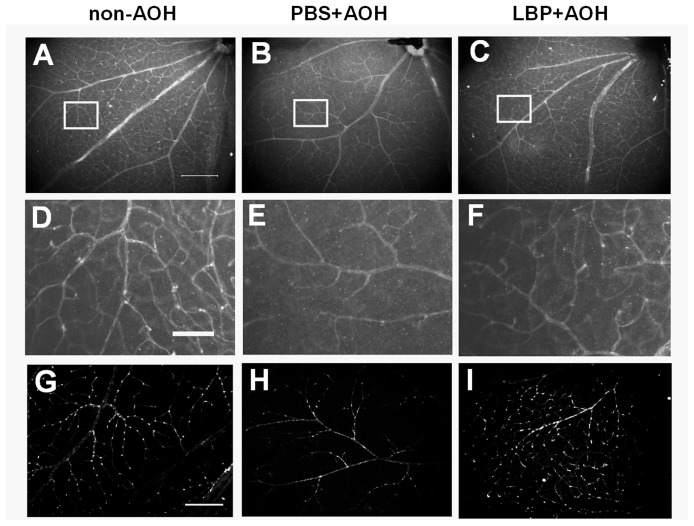
Alteration of retinal vasculature at day 4 after AOH treated with PBS and LBP. (A–C) Representative photos of the profile of PECAM-1-labeled retinal vasculature on retinal flatmounts, in the non-AOH control group, PBS-fed-AOH group and LBP-fed-AOH group. (D–F) The enlarged photos of white boxes in (A–C). (G–I) Representative photos of NG2-labeled pericytes in retinal vasculature on retinal flatmounts, in the non-AOH control group (G), PBS-fed-AOH group (H) and LBP-fed-AOH group (I). Scale bars: 200 µm in A–C and G–I; 50 µm in D–F. PECAM-1, Platelet endothelial cell adhesion molecule 1; NG2, neuron-glial antigen 2; PBS, phosphate-buffered saline vehicle; AOH, acute ocular hypertension; LBP, *Lycium Barbarum Polysaccharides* solution.

Using PECAM-1 to label blood vessel endothelial cells, the profile of retinal vasculature was shown on flat-mounts. Compared with the non-AOH control (Figure 5A, 5D), the density of retinal blood vessels in the PBS-fed-AOH group was reduced (Figure 5B, 5E). However, the density of retinal vessels in the LBP-fed-AOH group (Figure 5C, 5F) was similar to that in the non-AOH control retina. Also, on flat-mounted retinas, the density of NG2 labeled pericytes in the PBS-fed-AOH group was reduced (Figure 5H) when compared with the non-AOH control (Figure 5G). However, in the LBP-fed-AOH group (Figure 5I), the density was similar to the non-AOH control retina.

**Figure 6:**
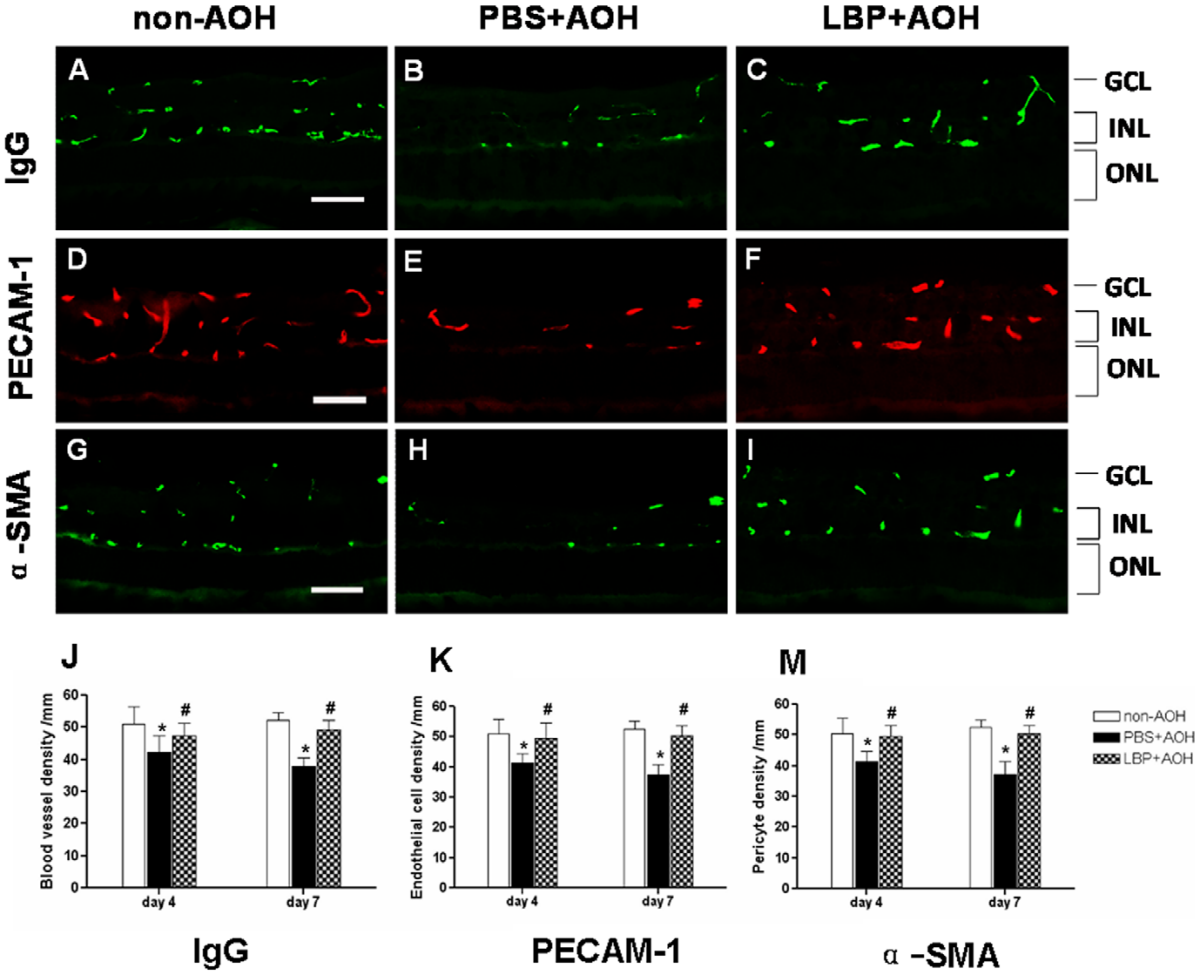
Attenuation of the density of retinal blood vessels after AOH treated with PBS and protection by the treatment of LBP. Representative photos of the IgG-labeled retinal blood vessels on retinal sections, in the non-AOH control group (A), PBS-fed-AOH group (B) and LBP-fed-AOH group (C) at day 7. Representative photos of the PECAM-1-labeled endothelial cells on retinal sections, in the non-AOH control group (D), PBS-fed-AOH group (E) and LBP-fed-AOH group (F) at day 7. Representative photos of the α-SMA-labeled pericytes on retinal sections, in the non-AOH control group (G), PBS-fed-AOH group (H) and LBP-fed-AOH group (I) at day 7. Quantification of the time course change (day 4 and day 7) of blood vessel density (J), endothelial cell numbers (K) and pericyte numbers (M). *P<0.05 compared with non-AOH; # P<0.05 compared with PBS+AOH. Scale bar: 50 µm. IgG, Immunoglobulin G; PECAM-1, Platelet endothelial cell adhesion molecule 1; α-SMA, α-smooth muscle actin; PBS, phosphate-buffered saline vehicle; AOH, acute ocular hypertension; LBP, *Lycium Barbarum Polysaccharides* solution; GCL, ganglion cell layer; INL, inner nuclear layer; ONL, outer nuclear layer.

To further quantify the change in blood vessels in the AOH retina, IgG immunostaining was used to evaluate the density of retinal blood vessels, PECAM-1 immunostaining was used to evaluate the density of endothelial cells and α-SMA immunostaining was used to evaluate the density of pericytes of blood vessels on retinal cross-sections. Since IgG immunostaining could be clearly detected inside the lumen of blood vessels, counting the labeled blood vessels indicated the number of blood vessels. This is different from the IgG extravasations described above which focused on signals outside the lumen of the blood vessels. Compared with the non-AOH control retina (Figure 6A), the density of IgG-labeled blood vessels in the GCL and INL of the PBS-fed-AOH group was severely reduced (Figure 6B). Interestingly, in the LBP-fed-AOH group (Figure 6C), more IgG-labeled blood vessels were observed than the PBS-fed-AOH group, and this was comparable to the non-AOH control. Quantification supported these changes with statistical significance (Figure 6J).

PECAM-1 stained signals showing the density of blood vessel endothelial cells was decreased in the PBS-fed-AOH group (Figure 6E) when compared with the non-AOH control (Figure 6D), but it was higher in the LBP-fed-AOH group (Figure 6F) than the PBS-fed-AOH group, which was comparable to the density of the non-AOH control. Quantification supported these changes with statistical significance (Figure 6K). These data suggested that LBP treatment protected the endothelial cells of blood vessels against AOH.

Immunoreactivity of α-SMA staining also showed this trend of change (Figure 6G versus 6H and 6I) and quantification supported it with statistical significance (Figure 6M), which suggested that LBP treatment also protected the pericytes of blood vessels against the insult of AOH.

### LBP down-regulated the expression of ET-1

**Figure 7:**
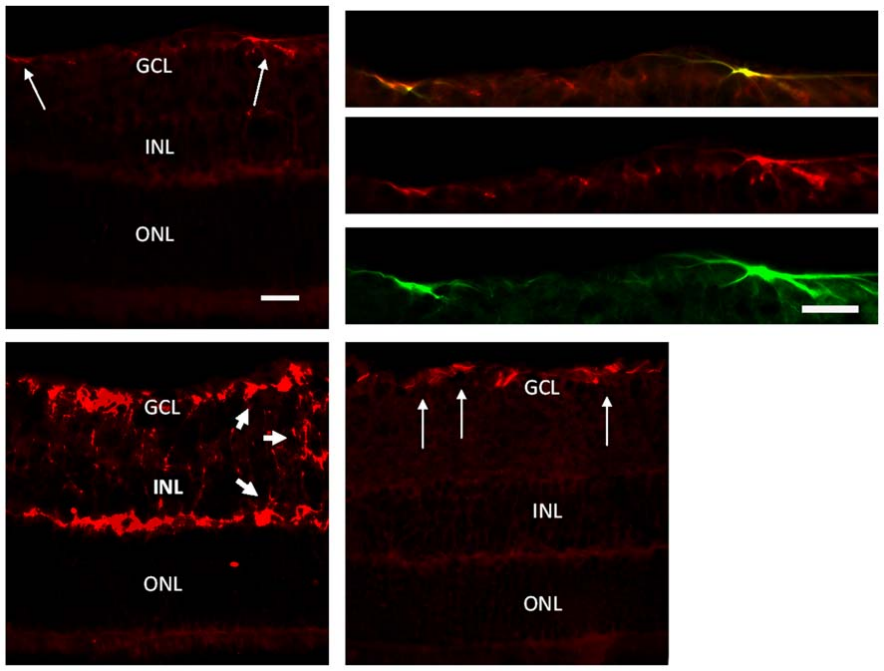
Up-regulation of ET-1 in PBS-fed retina and down-regulation in LBP-fed retina at day 4 after AOH. (A) The expression of ET-1 in the non-AOH retina. (B–D) Co-localization of ET-1 in astrocytes in GCL (yellow color) in (B) merged by the staining of ET-1 (red color) of (C) and GFAP (green color) of (D). (E) The distribution and expression patterns of ET-1 in the PBS-fed-AOH retina. (F) The distribution and expression patterns of ET-1 in the LBP-fed-AOH retina. Note that the expression of ET-1 in astrocytes (long white arrows) and in the processes of Müller cells (short white arrows). Scale bars: 20 µm. ET-1, endothelin-1; GFAP, Glial fibrillary acidic protein; PBS, phosphate-buffered saline vehicle; AOH, acute ocular hypertension; LBP, *Lycium Barbarum Polysaccharides* solution; GCL, ganglion cell layer; INL, inner nuclear layer; ONL, outer nuclear layer.

Endothelin-1 (ET-1) can contribute to the damage of BBB in ischemic brain as reported previously [9], [10]. In the present study, the expression of ET-1 was shown as a background staining in the non-AOH control retina with some locally strong staining signals in the GCL (Figure 7A–7D). These ET-1 positive signals (Figure 7C) belonged to astrocytes (Figure 7B) since they co-labeled with GFAP (Figure 7D). In the PBS-fed-AOH group, the expression of ET-1 was up-regulated in the GCL, IPL, INL and OPL (Figure 7E). In the LBP-fed-AOH group (Figure 7F), the expression of ET-1 was down-regulated when compared with the PBS-fed-AOH group; however, there were still stronger ET-1 positive signals in the GCL when compared with the non-AOH control retina, which should belong to the proliferated astrocytes as described above.

### LBP down-regulated the expression of RAGE

**Figure 8:**
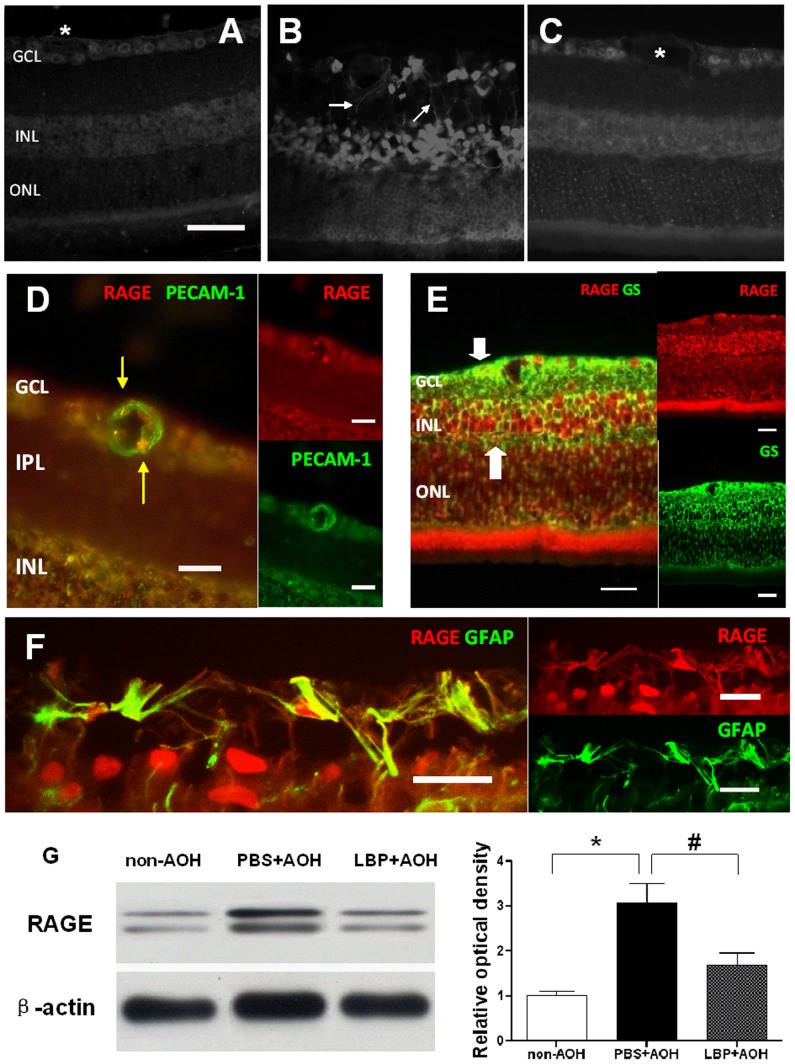
Up-regulation of the expression of RAGE in PBS-fed retina and down-regulation in LBP-fed retina at day 4 after AOH. Representative photos of RAGE immunostaining on retinal sections in the non-AOH control group (A), PBS-fed-AOH group (B) and LBP-fed-AOH group (C). The expression of RAGE is present in the processes of Müller cells (white arrows) in (B), and no RAGE signals are observed in blood vessels (asterisks) in (A) and (C). (D) The PBS-fed-AOH retina shows the co-localization of RAGE in endothelial cells (yellow arrows) using the marker PECAM-1 (green color) double-staining with RAGE (red color). (E) The PBS-fed-AOH retina shows the co-localization of RAGE is also present in the end-feet, processes and cell bodies of Müller cells (bold white arrows) in yellow color using the marker GS (green color) double-staining with RAGE (red color). (F) The PBS-fed-AOH retina shows the co-localization of RAGE is also present in astrocytes (yellow color) using the marker GFAP (green color) double-staining with RAGE (red color). (G) Comparison of RAGE level in retinas from the non-AOH group, PBS+AOH group and LBP+AOH group. Scale bars: 20 µm. * P<0.05 compared with non-AOH; # P<0.05 compared with PBS+AOH. RAGE, the receptor for Advanced Glycation Endproducts; PECAM-1, Platelet endothelial cell adhesion molecule 1; GFAP, Glial fibrillary acidic protein; GS, Glutamine synthetase; PBS, phosphate-buffered saline vehicle; AOH, acute ocular hypertension; LBP, *Lycium Barbarum Polysaccharides* solution; GCL, ganglion cell layer; IPL, inner plexiform layer; INL, inner nuclear layer; ONL, outer nuclear layer.

RAGE was detected in the clinical patients of glaucoma [36]. It was reported that one role of RAGE was to transport Aβ across the BBB to the brain tissue in Alzheimer's disease [13]. In the present study, a mild positive staining of RAGE in the neurons of the GCL and INL was observed in the non-AOH control retina (Figure 8A). In the PBS-fed-AOH group, the expression of RAGE was up-regulated; the strong positive signals were found in the neurons of the GCL and INL (Figure 8B). Co-labeled with PECAM-1, the positive signals of RAGE were also observed in endothelial cells (Figure 8D). The positive signals of RAGE were co-labeled with Glutamine synthetase (GS) and found in the end-feet, processes and the cell bodies of Müller cells (Figure 8E). Astrocytes in the GCL labeled with GFAP could also express RAGE (Figure 8F). In the LBP-fed-AOH group, the expression of RAGE was down-regulated when compared with the PBS-fed-AOH group showing no positive signal in the blood vessel endothelial cells; however, there were still positive signals in the neurons of the GCL and INL (Figure 8C). Western blotting data confirmed the increased level of RAGE in the PBS-fed-AOH retinas when compared with the non-AOH control and the decreased level in the LBP-fed-AOH retinas when compared with the PBS-fed-AOH group (Figure 8G).

### LBP down-regulated the expression of Aβ_1–42_

**Figure 9:**
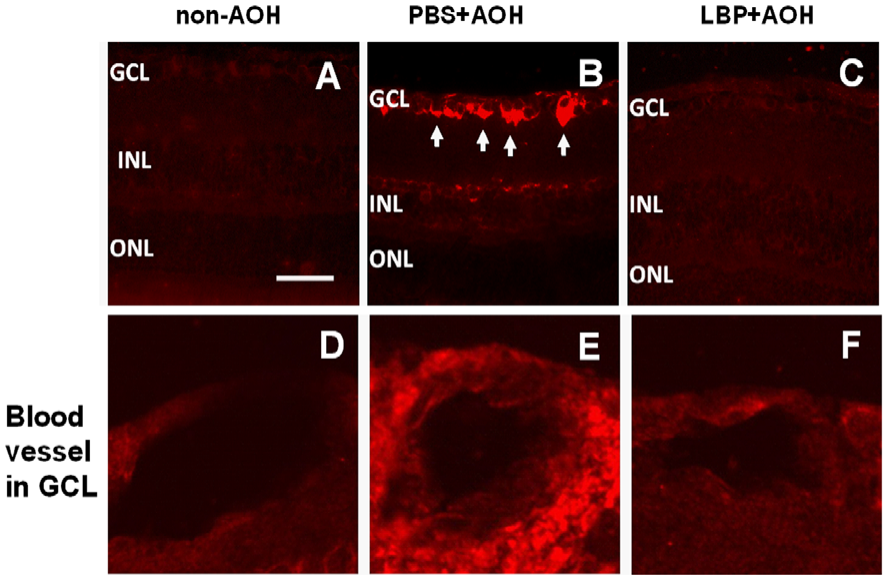
Up-regulation of the expression of Aβ_1–42_ in PBS-fed retina and down-regulation in LBP-fed retina at day 4 after AOH. Representative photos of Aβ_1–42_ immunostaining on retinal sections in non-AOH control group (A), PBS-fed-AOH group (B) and LBP-fed-AOH group (C). Note that the Aβ1–42 staining is present in neurons of GCL (arrows) in (B). (D–F) The enlarged blood vessel in GCL from the non-AOH, PBS+AOH and LBP+AOH group, respectively. Scale bars: 50 µm. Aβ_1–42_, amyloid-beta 1–42; PBS, phosphate-buffered saline vehicle; AOH, acute ocular hypertension; LBP, *Lycium Barbarum Polysaccharides* solution; GCL, ganglion cell layer; INL, inner nuclear layer; ONL, outer nuclear layer.

The expression of Aβ_1–42_ was detected in the chronic ocular hypertension rats and in DBA/2J glaucoma mice [37], [38]. In the present study, the non-AOH control retina did not express Aβ_1–42_ (Figure 9A, 9D); however, in the PBS-fed-AOH group, staining of Aβ_1–42_ were detected in: 1) the neurons in the GCL, 2) the INL but the signal was patchy (Figure 9B), and 3) the lumen of blood vessels (Figure 9E), suggesting the presence of the vascular source of Aβ_1–42_ deposition in AOH. However, in the LBP-fed-AOH group, the positive staining signal of Aβ_1–42_ was rarely detected (Figure 9C, 9F).

### LBP down-regulated the expression of AGE

**Figure 10:**
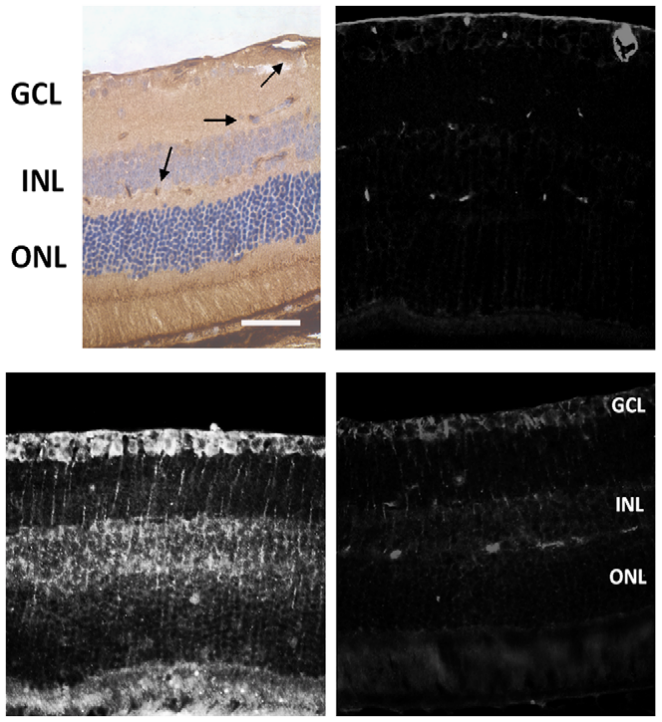
Alteration of the expression of AGE in PBS-fed retina and LBP-fed retina at day 4 after AOH. (A) The DAB staining shows the distribution of AGE in non-AOH retina. Note that the arrows showing the positive signals of AGE are located in blood vessels. (B) The corresponding fluorescence staining of AGE in non-AOH retina. (C) The expression patterns of AGE in PBS-fed-AOH retina. (D) The expression patterns of AGE in LBP-fed-AOH retina. Scale bar: 50 µm. AGE, Advanced Glycation Endproducts; PBS, phosphate-buffered saline vehicle; AOH, acute ocular hypertension; LBP, *Lycium Barbarum Polysaccharides* solution; GCL, ganglion cell layer; INL, inner nuclear layer; ONL, outer nuclear layer.

Since AGE is a key ligand of RAGE and was also detected in glaucomatous retina from patients, we also used it to examine the effect of LBP on AOH retina. In the non-AOH control retina, the positive AGE immunoreactivity was confined to the blood vessels (Figure 10A, 10B). In the PBS-fed-AOH group, the strong AGE immunoreactivity was expressed in the neurons in the GCL and INL and the processes of Müller cells in the IPL associated with their end-feet in the ILM and OLM; even some non-shaped diffuse staining signals were detected distributing in the parenchyma of other retinal layers (Figure 10C). In the LBP-fed-AOH group, the expression of AGE was down-regulated compared with the PBS-fed-AOH group; the positive immunoreactivity was mostly confined to the blood vessels. However, there were still some strong positive signals in the end-feet and processes of Müller cells (Figure 10D).

## Discussion

In this study, we demonstrated transient elevation of IOP at 90 mmHg for 60 minute induced a marked loss of RGCs and thinning of inner retinal layers, which are associated with retinal gliosis and retinal vasculature damage. Interestingly, we found that feeding LBP 7 days before AOH procedure improved the survival ratio of RGCs and reduced the thinning of inner retinal layers. In addition, blood vessel cells were protected and eventually the BRB damage was prevented after AOH insults.

Combined with the ischemic and mechanical injury, AOH model shares some similarity with clinical acute glaucoma. It has thus been commonly used to investigate the degeneration and neuroprotection for RGCs [31], [39] [40]. In the previous studies, according to their specific investigating designs, the elevation of IOP and its duration has been regulated correspondingly, which are different from each other. The common range of IOP is from 80 to 120 mmHg and the duration of AOH is from 45 to 120 min [41] [31]. One previous study of electroretinography (ERG) has reported that nonspecific functional changes will be produced when AOH is above 50 mmHg in Brown-Norway rats. Thus, level below 50 mmHg belongs to experimental glaucoma model [42]. However, another study reports under the condition of 80–90 mmHg with the duration of AOH 120 min to Lewis rats and 90 min to C57BL/6J mice could produce the similar capillary degeneration like diabetes [41]. Taken together, from the view of vasculature, the elevation of IOP and extension of its duration could induce damage to blood vessels to initiate the mechanism of ischemia reperfusion (I/R) injury. Clinical studies have shown that transient elevation of IOP higher than 30 mmHg during acute episode of glaucoma could induce I/R injury in human [43], [44], [45]. Thus, in AOH animals, it might involve two critical mechanisms of ischemic and mechanical injury under the above range of experimental ocular hypertension.

Previous study has reported the loss of capillaries in the AOH model using the method of retinal vasculature isolation [41]. Here, we verified this result by counting the IgG stained blood vessels. Although using the methods in this study, we could not observe directly the degenerated capillaries, being represented as a cellular capillaries lacking perfusion [34], [35], [41]. Our data could show the reduction of retinal vasculature in the PBS-fed-AOH group and the increase in the LBP-fed-AOH group. Although we did not observe and compare the nonvascular cells in the AOH retina between the PBS fed group and LBP fed group, which was present under ischemic conditions as reported in other models [34], [35], [41], our data showed the protective effect of LBP to increase the survival of retinal endothelial cells and pericytes from the AOH injury. Moreover, since pericytes could have provided vascular stability and controlled endothelial proliferation [35], it is reasonable to believe that LBP protected pericytes. Therefore, LBP was effective in resisting endothelial proliferation and keeping the stability of their numbers in ischemia. The interesting findings from our study were to show protective effects of LBP on retinal blood vessels. As retinal ischemia is a common pathology in many eye diseases such as glaucoma, diabetic retinopathy and other vascular diseases of retina, our data suggests that LBP might be a potential useful drug for treating these similar diseases.

BRB disruption has been reported in other ischemic models [9], [46]. Here, we found that it was also severely damaged in AOH retinas, and LBP treatment maintained the integrality of BRB, which is consistent with our previous study of MCAO retina recently [11]. Thus, the protective effect of LBP on BRB in AOH retinas may be related to the survival of blood vessel endothelial cells and pericytes associated with the integrality of tight junction between endothelial cells. The expression level of occludin has been reported to decrease in ischemia [9], [10]. Moreover, our previous study also has shown that over-expression of ET-1 in astrocytes (GET-1 mice) and in blood vessel endothelial cells (TET-1 mice) could contribute to the damage of blood-brain-barrier (BBB) by down-regulating the level of occludin in the brain of MCAO [9], [10]. Here, we demonstrated the up-regulation of ET-1 in AOH retinas, and down-regulation after LBP treatment. Thus, the effect of LBP on down-regulating the expression of ET-1 in AOH retinas could be another mechanism for its protective effects on BRB damage.

The significance of BBB damage is extravasation of plasma protein from the circulation, which can trigger inflammation, oxidative stress, perivascular edema, and axonal demyelination [47]. Moreover, alterations in BBB could facilitate some cross-membrane transportation for circulating toxins [48], [49]. Assuming this is also true for BRB, the extensive distribution of IgG extravasations in the present study suggested the presence of toxic factors effusing from the disrupted BRB into the AOH retina. Thus, in the AOH retina, the disruption of BRB may be served as a basis for the involvement of damaging factors in the molecular mechanisms of retinal degeneration. Subsequently, protection to BRB damage should be a key target for neuroprotection related to ischemia.

The present study demonstrated the expression and distribution patterns of the receptor for advanced glycation end products (RAGE) in the AOH retina. Associated with the alternation of BRB, vascular RAGE could serve as a cross-membrane transporter for the deposition of Aβ in tissue. Here, we also detected Aβ_1–42_ immunoreactivity in blood vessels and neurons in the AOH retina. Such molecular mechanism has already been demonstrated in an experimental AD animal model [13]. The direct effect of this transportation is the increasing release of ET-1 from endothelial cells, which could in turn contract the blood vessels to promote ischemia [13]. It also may be a cause of the up-regulation of ET-1 detected in the AOH retina.

Previous study has reported the accumulation of advanced glycation end products (AGEs) in ischemic brain [50]. Our present data confirmed that AGE was up-regulated in the AOH retina by immunohistochemical staining. Furthermore, we also detected the alteration of the distribution of AGEs after AOH from blood circulation to the AOH retina. We believe that it may be related to the up-regulation of vascular RAGE. The transportation of AGEs by vascular RAGE could be mediated by similar mechanism for the transportation of circulating Aβ [13]. One of such negative effects is to initiate the reactivation of AGE-RAGE axis in the AOH retina [51], [52]. One is contributing to the disruption of BRB [53], as a cause of BRB damage as observed in this study. Other negative effects include inducing pro-inflammatory, pro-adhesive and growth-stimulating signals [54]. Moreover, previous study has reported the up-regulation of expression of AGE and RAGE in the retina of glaucoma patients [36], as well as Aβ in DBA/2J glaucoma mice [37]. Taken together the presence of these biomarkers in the AOH retina observed in our study, it is suggested that RAGE activity may be responsible for the accumulation of AGE and Aβ, and ischemia might be one of the initiator of reactivation of RAGE in glaucoma.

LBP could protect cerebral neuron from Aβ toxicity as reported in our previous *in vitro* studies [18], [19], [20], [21]. Here, we verified its neuroprotective effect of anti-Aβ_1–42_ in a vivo model of AOH. The possible molecular mechanism is related to reducing the transportation of Aβ by vascular RAGE from circulation. Furthermore, in the present study, we also demonstrated that LBP could suppress the reactivation of AGE-RAGE axis, as known in many ischemic related retinopathies, which could be interesting in the protective mechanisms of BRB and retina in diseases.

### Conclusions

Our data evaluated the neuroprotective effects of LBP to anti-ischemic insult induced by AOH. The possible mechanism is related to down-regulating the expressions of RAGE, ET-1, Aβ and AGE in the retina, as well as the related signaling pathways leading to inhibiting the vascular damage and the neuronal degeneration in AOH. The present study suggests LBP could prevent damage to RGC and retinal neurons from ischemic injury; therefore, by targeting the mechanism of blood vessel protection, LBP would also be a potential treatment for vascular-related retinopathy.
